# A review of animal health and drug use practices in India, and their possible link to antimicrobial resistance

**DOI:** 10.1186/s13756-020-00760-3

**Published:** 2020-07-08

**Authors:** Florence Mutua, Garima Sharma, Delia Grace, Samiran Bandyopadhyay, Bibek Shome, Johanna Lindahl

**Affiliations:** 1 International Livestock Research Institute, P. O. Box 30709, Nairobi, 00100 Kenya; 2 Zoonoses Science Centre, Uppsala University, P. O. Box 70790, SE 750 07 Uppsala, Sweden; 3 Indian Veterinary Research Institute, Eastern Regional Station, 37 Belgachia Road, Kolkata, 700 037 India; 4 National Institute of Veterinary Epidemiology and Disease Informatics, Bangalore, India; 5 Department of Clinical Sciences, Swedish University of Agricultural Sciences, P. O. Box 70790, SE 750 07 Uppsala, Sweden

**Keywords:** Antimicrobial use, Antimicrobial resistance, Animal health, Theory of change

## Abstract

**Background:**

Livestock production, particularly the dairy sector, is important for food and nutritional wellbeing of communities in India, it supports livelihoods of many farmers, and contributes to the economy of the country. India is a high consumer of antibiotics and antimicrobial resistant (AMR) bacteria are a major public health concern.

**Objectives:**

Our objectives were to identify animal health and drug use practices that may contribute to emergence and spread of AMR in the country, review previous AMR- mitigation strategies, and discuss “theory of change” as an approach to informing the choice of interventions.

**Methods:**

We undertook a desk review of literature to identify practices with potential to contribute to emergence and spread of antimicrobial resistance in India. Searches were done in PubMed, Google scholar, and Google. Data were synthesized and discussed by themes.

**Results:**

Animal disease surveillance is less developed and infrastructure to support delivery of services is inadequate. Several groups are known to offer animal health services. The untrained “animal health workers” and para-veterinarians are more popular with farmers as they charge less for consultations (compared to veterinarians who are few and charge more). Over-the-counter access of antibiotics, without prescription, and direct marketing of drugs to farmers are common. Because of this, farmers are able to treat their animals and only consult when cases become non- responsive to treatment. Antibiotics are mostly used in management of mastitis cases. Drug withdrawal periods are rarely observed and occurrence of antibiotic- contaminated milk has been reported. Awareness on AMR is low and antimicrobial stewardship in livestock is yet to be developed. Initiatives such as the National programme for containment of AMR, National Action Plan on AMR, and the National Health policy point to government’s commitment in addressing the problem of AMR in the country.

**Conclusion:**

Several animal health and drug use practices, with potential to cause AMR, have been described, and their contribution can be discussed further by engaging stakeholders in a “theory of change” exercise. Interventions that address AMR from the animal health perspective should be promoted, and incentives to increase their adoption explored.

## Introduction

Antimicrobial use (AMU) is a driving force for antimicrobial resistance (AMR) in animal husbandry [1]; AMR occurs when a microorganism no longer responds to a drug to which it was originally sensitive [2]. Over-prescription of drugs, use of too high or too low dosages, and incorrect duration of medication [3] can aggravate the problem. Attempts to respond to the rising food demand, driven by high population growth, urbanization, and rising income, have put pressure on farmers to produce more but in limited spaces [1, 4, 5].

Antibiotics are also included in animal feeds. They are added in small (sub-therapeutic) doses to promote growth, however, their addition in animal feeds can contribute to development of AMR bacteria [6, 7]. The use of growth promoters has been banned in many countries including the European Union. There have been recommendations to regulate their use in India [4, 8, 9] particularly for colistin given its use in human health and the risk of AMR. The global consumption of antimicrobials in food animals was estimated at 63,151 tons (±1650) tons in 2010 and at 131,109 tons in 2013 [10, 11]. The use antimicrobial in intensive production systems is high [12] and explains about 34% of the global increase in consumption [10]. A global rise of up to 200,235 tons by 2030 has been projected [11]. India accounts for about 3% of the global consumption of antimicrobials in food animals [10].

Antibiotics are also widely used in treatment of sick animals. For food animals, failure to observe drug withdrawal periods results in products that are contaminated [13] and raises questions on their safety when consumed by humans. Maximum residue levels of antibiotics have been established, over which products are deemed unfit for consumption, and should be discarded (in a manner that minimizes the risk of environmental contamination) [14, 15]. Antimicrobial resistant bacteria can be transferred from food animals to humans either through direct contact with animals, contaminated foods, or indirectly through contaminated environments [1, 16, 17]. Consequences of infection with AMR bacteria include long hospital stays, increased mortalities, loss of protection for patients requiring surgery, and increased treatment costs [2].

Antimicrobial resistance is a complex problem and tackling it is challenging, however, there are on- going efforts towards its containment [18]. Attempts to improve antibiotic use should first identify key factors that contribute to their inappropriate use and, where possible, consider interventions that are specific to solving the identified problems [3]. Because antimicrobial resistance affects health of humans, animals, and the environment, “One Health” mitigation approaches, with involvement of relevant sectors, are required [19]. The Global Action Plan by the World Health Organization (WHO) outlines a number of strategies that countries could use to mitigate the increasing risk, including “improving awareness and understanding of AMR through effective communication, education and training” [20]. India is a hotspot for antimicrobial use [5, 21] and a prior knowledge of factors that contribute to their increased use (including their prioritization) can guide the choice and design of AMR intervention strategies. Although a number of studies have examined resistance profiles of bacterial isolates, data on antibiotic use (and reasons for use) are still limited [4] and this makes it difficult to design appropriate packages. Further, a review of previously suggested strategies can help identify those that can be piloted or scaled out, based on factors such as their efficacy, ease of use and cost. A Theory of Change (ToC) approach improves our understanding of how change occurs [22] and outlines what needs to be done to achieve desired outcomes. This is important given that a number of AMR- related activities are currently being undertaken (or are planned), by different entities (research, policy etc.), and to address different objectives (evidence generation, scaling- out of interventions including capacity development.). Theory of Change can also inform identification and monitoring of interventions to combat AMR. Objectives of this study are to 1) identify animal health and drug use practices that contribute to development of drug resistance in smallholder dairy systems of India 2) review previous and on-going AMR- mitigation strategies and 3) discuss the ToC as an approach to informing the choice of interventions to better address the problem of AMR.

## Methodology

To achieve the three objectives, a desk review of literature was done to identify practices with potential to contribute to emergence and spread of antimicrobial resistance in India (Table 1). Searches were done in PubMed, Google scholar, and Google. An appropriate combination of key words was used, including: Animal health problems (or animal husbandry, agriculture); Antimicrobial use (or drug/ antibiotic use, AMU); and Antimicrobial resistance (or drug resistance, antibiotic resistance, AMR). Articles cited in the reviewed publications were also sought and reviewed. Our focus was on practices in the dairy sub-sector. Content found to be relevant for the study was extracted. The findings were synthesized and described by themes; the dairy sector and the problem of mastitis, delivery of animal health services, practices pertaining to antibiotic use, antibiotic residues in milk, AMR interventions, antimicrobial stewardship, and “theory of change”. This was done by the first author and reviewed severally by the authors.

**Table 1:** Practices related to antibiotic use in animal health, and their implications in antimicrobial resistance

	Animal health practices	Implications
Farm practices (with potential to cause AMR) *Chauhan* et al. [23]*; Kumar and Gupta* [24]	Selling of milk from cows given antibiotics	In cases where withdrawal periods have not been observed and residue levels are beyond the recommended levels, consumers can be exposed to low antibiotic doses, which can result to resistant bacteria.
	Inadequate disease-control practices including vaccination	Disease control is important as new infections are avoided and the need to use antimicrobials is reduced. The risk of AMR is minimized.
	Not aware about antibiotic withdrawal periods, for those aware, considering it impractical given the loss implications	Farmers are likely to sell antibiotic-contaminated milk, and this has serious health implications
Unrestricted access to antibiotics *Chauhan* et al. [23]*;* *Kumar and Gupta* [25]*; Chauhan* et al. [26] *Bhushan* et al. [27]	Direct marketing of drugs to farmers	The strategy may encourage farmers to use antibiotics in cases where they are not required. Prudent use of drugs is important in addressing the problem of AMR.
	Over-the-counter access (informal prescribers, with or without prescription, and through re-use of old prescriptions).	Inappropriate use is promoted.
	Use of low-cost antibiotics by small- scale farmers (how much is used depends on the severity of infection)	A problem if these are of poor quality or are easily available over- the-counter as there is tendency to use them inappropriately. Exposure to low doses over a long period of time may encourage selection of resistant bacterial strains.
	Farmers administer antibiotic to animals irrespective of whether the disease is infectious or not	This implies misuse of antibiotics and may trigger AMR.
	Use of antibiotics labelled for humans (and those for other livestock species)	Appropriate dosages and withdrawal period cannot be determined. Use of last-resort antibiotics would have serious health implications.
Consultation when an animal is sick. *Garg and Mohanta* [28]*; Chauhan* et al. [23]*; Kumar and Gupta* [24]	Consulting veterinarians after the case has become serious, and often after sick animals have been treated by unqualified individuals	Chronic cases are less likely to be successful, and the infection may have become resistant, making the veterinarians unable to save the animal, and the farmer loses confidence.
	Consulting with unprofessional groups (e.g. milk vendors and the para-veterinarians)	They are not trained and therefore not aware of the right medication to use. They are also not knowledgeable about AMR.
	Lack of operational laboratory facilities (lack of microbiologists, equipment etc.).	Quality tests allow for confirmation of specific pathogens and will inform the choice of antibiotics to use. Tests are also important in surveillance of AMR.

## Results

### The dairy sector and the problem of mastitis

About 70% of India’s population is engaged in agriculture and livestock keeping [29]. India has the highest number of dairy animals in the world including about 300 million bovines [30, 31]. Milk production is by buffaloes (56% of total milk production) [32] and dairy cows, depending on the region. The animals are mostly kept by small-scale farmers [33], and according to Kurup 2001 [34], also mentioned in the FAO [35] report on *“impacts of mastitis in small scale dairy production systems”*, these farmers own over 60% of all milk animals in the country. Few farmers (about 5%) own more than 5 animals [36]. The per capita milk consumption vary across states but is said to be high in urban areas [37]. About 50% of the milk is consumed at the farm level [33]. Cost and quality are issues in the sector [38].

Mastitis remains a problem in many dairy herds with about 70% of all losses being perceived to be due to the infection [32, 39]. Several factors contribute to its occurrence, among them the failure to disinfect cow sheds, incomplete and unhygienic milking, and inability to isolate sick animals [40–42]. Foodborne bacterial agents including *E. coli*, *Proteus* spp., *Klebsiella* spp., *Staphylococcus aureus, Streptococcus spp,* and *Corynebacteria spp* have been reported in India [32, 40, 43]. Milk found to be contaminated with these pathogens is unfit for human consumption and should be discarded. A meta-analysis study by Bangar et al. [44] reported a pooled sub-clinical mastitis prevalence of 46% in India (using 6344 cows from 25 studies). Another review by Krishnamoorthy et al. [45] reported prevalence estimates of 41% (*n* = 25,455; subclinical mastitis) and 27% (*n* = 6978; clinical mastitis). Farmers will want to initiate mastitis therapy early enough to minimize losses [46] and this may include using broad-spectrum antibiotics [23, 47, 48]. Medication is mostly delivered into the cow’s udder [13] (although injections may be applied in a few of the cases). Das et al. [49] analysed losses due to mastitis in Odisha, India, and reported a milk loss of about 9.9 l per day per farm (which locally translated to INR 297 per day). Jingar et al. [50] estimated an average loss of INR 1227 for clinical mastitis in cross-bred cows (which included production and treatment losses).

### Delivery of animal health services

India has about 34,500 field veterinarians (against a required number of 75,000) [51]. Majority of the staff (over 75%) are involved in veterinary care; about 3.5% are attached to disease control and investigation programs [52]. There are 13 state veterinary universities, 58 veterinary colleges, and about 21 public veterinary vaccine production units [4, 51]. Animal health and disease control services are provided by the state [51], but in spite of these being heavily subsidized, they are still not adequate to serve the needs of poor farmers [53] (who may instead choose to consult untrained individuals). There are villages with no access to veterinary dispensaries [54, 55], surveillance systems fail to reach all parts of the country [23, 51], and many remote areas are not included in the on-going programmes. In addition, available laboratory facilities are not adequately equipped and veterinarians rarely depend on them to manage diseases [23]. Antimicrobial susceptibility testing is only performed where failure or low response to initial therapy is observed (and therefore not considered in routine diagnosis of cases) [56]. In most cases, samples are submitted by the farmers themselves (mainly the large-scale ones who may have been directed to do so by their veterinarians) [23, 56].

The main animal health providers are veterinarians, para-veterinarians (para-vets, or colloquially known as *pranibandhu* or *pranimitra*), and the untrained “quacks” [56]. A “quack”, according to Ali et al. [57] is *“an unqualified person who claims publicly to have a medical knowledge and skill which in fact he or she does not have”*. Para-vets have basic training in animal health and work mostly in government hospitals [53]. Veterinarians are few in number and their busy schedule may limit their participation in outreach activities [23, 25, 56]. They are, at times, impelled to give advice and prescription over telephone calls [23]. For management of refractory mastitis, veterinarians prefer to use new generation antibiotics as these are known to provide positive treatment outcomes. The drugs are however expensive and farmers may not afford to pay for them [36], but most importantly, they may be the last resort for critical infections in humans, and using them may necessitate selection for resistance, thus limiting their future use in human health. In attending to sick animals, field veterinarians may follow the advice of influential persons [23], who are not be trained and will likely not be aware of AMR and its public health implications.

### Practices pertaining to antibiotic use

Smallholder dairy farmers rarely use antibiotics for prophylaxis and animal vaccination is not routine [24, 56]. Extension systems are not fully developed [23] and farmers are the ones who decide on when to use antibiotics [48]. Sick animals are not isolated, and are at times treated by the farmers themselves (without consulting trained animal health professionals) [23, 28, 41]. It is easy to access antibiotics over-the-counter [21, 24, 26, 58], without prescriptions or even with old ones [23, 58]. For the treatments, farmers prefer medicines that give them quick results, and based on what is available, their previous experience with the drug while managing similar symptoms, and advice from veterinarians, feed stores, and peers [23, 24]. Kumar and Gupta [24] reported increases in drug dosages in cases where response to therapy was perceived to be poor. Antibiotics are also used to cover animals after parturition and to increase milk production [24, 38]. The other problem is the direct marketing of drugs to farmers by salesmen of drug distributors who are said to have a strong presence in the communities [23, 26]. Access of drugs through milk vendors, feed stores, and cooperatives has also been reported [24]. It is not uncommon to see critically important antibiotics being used in animals and aquaculture [27]. In addition, farmers are unaware of the consequences of improper disposal of milk from sick and treated cows [59] which may contribute to environmental contamination.

Distance from animal health centres, ownership of crossbred cattle, as well as the financial position of the farmer does influence the choice of animal health providers [53]. Mirajkar et al. [55] analyzed preference for veterinary service delivery in a sample of farmers in Sangli District, Maharashtra State, India. The findings varied by block, in some, the smallholder farmers sought services from private veterinarians (who were considered more available whenever called on by the farmers); others sought services either from government officials or through cooperatives where they were members. In Mizoram, farmers were found to rely more on para-vets (63%; *n* = 100) than on professionals [60]. Farmers in Churu District consulted quacks first (82%) before seeking advice from veterinarians [61]. The word “private doctors” refers to informal drug prescribers [23]; Bardhan et al. [53] calls them “private veterinary practitioners”. They charge less and are perhaps the main service providers at the farm level; smallholder farmers have a tendency to consult veterinarians only after the case has become serious and after the animal has been subjected to multiple antibiotics [26, 28, 50]. Also, farmers rarely share previous treatment history with their veterinarians [56] and this makes it difficult to determine what treatment option to use to minimize the risk of AMR. Compared to small-scale farmers (who prefer to consult paraprofessionals), large-scale dairy farmers are more likely to seek the services of qualified veterinarians and also likely to use antibiotics prudently [24]. Findings from the study by Patel et al. [62] indicate “veterinarians only” (28%), “quacks only” (22%), and both “veterinarians and quacks” (49%) as providers of animal health services. Similarly, small-scale farmers (*n* = 56) studied by Kumar and Gupta [24] relied on veterinarians (50%), para-veterinarians (30%), over-the-counter sales (12%) and milk vendors (7%). Farmers may choose to sell animals that fail to respond to treatment [59].

Chauhan et al. [26] identified cost as a deterrent in seeking professional veterinary services in Ludhiana, Guwahati and Bangalore. It is also the reason why some farmers will opt to consult other value chain actors [24] and not the qualified professionals. High cost of animal treatment was also reported by dairy farmers in Haryana [25]. In the study by Chauhan et al. [23], farmers said they could not afford to pay for veterinarians to visit their farms, and therefore chose quacks who charged them less. They may, at times, request veterinarians to prescribe low-cost antibiotics [24], which, except for complicated cases, may easily be granted. Increased use of antibiotics is a risk for AMR [63]. The use of traditional herbs in animal health (mostly by smallholders) in treatment of multiple health problems has been reported [64, 65]. Varshney and Naresh [66] investigated the efficacy of homeopathic medicine in treatment of acute mastitis, and reported an overall effectiveness of 86.6%, a recovery period of 3–28 days, and a cost estimate of US$0.47 (compared to a cure rate of 59.2%, a recovery period of 2–15 days, and a cost of US$3.28 observed in the antibiotic- treated group). New formulations are also gaining popularity among dairy farmers [66]. A strong reliance on traditional medicine can hinder farmer participation in agricultural extension [23] especially in programs meant to promote use of modern medicine.

### Antibiotic residues in milk

The study by Parkunan et al. [59] point to addition of antibiotics to feed and water meant for animals, by farmers. Antibiotic residues are found in animal products shortly after treatment, and the way to avoid them is to wait for a prescribed period, the withdrawal period, to be over, before an animal is slaughtered, or milk is taken for consumption. It varies with the type of the drug. There are two main reasons why farmers are not adhering to this. One reason is likely the lack of knowledge; farmers are sometimes not aware that they are using antibiotics, particularly if it is in the feed, and even if they know it, many are not aware about the importance of the withdrawal period and its link to public health [67]. The second reason is financial, where farmers know that they should not use the milk but cannot afford not to do it. For mastitis, they fear losing milk (and therefore income) if treatment is initiated [46]. Dairy farmers are often advised to exclude treated animals from the milk supply chain for a specific period of time [13], as indicated in the label, to allow for the residues to fall to the required level. Similarly, when an animal is not responding to treatment, farmers may consider selling it for slaughter as a way of minimizing the loss. AMR can greatly affect animal health and production [68].

In India, the use of antibiotics to manage mastitis cases is common [36], several drugs are used, either singly or in combination [13]. Contamination of milk with antibiotic residues has been reported in several studies (Table 2). Contamination with oxytetracycline residues was reported by Sudershan and Bhat [73]. The CDDEP [4] report makes references to a regulation on withdrawal period with a 28-day period being used where this is not specified. Milk from cows treated with antibiotics is either fed to calves, sold for human consumption, or disposed [24, 59].

**Table 2:** Results of a desk review exercise to determine the status of antibiotic residues in milk in India

Study authors	Study area	Test analyses procedures (number of samples)	% Positive
Kalla et al. [47]	Selected coastal districts of Andhra Pradesh	Delvo test (*n* = 300 raw milk)	7 (penicillin); 5 (tetracycline); 6 (oxytetracycline)
Kumarswamy et al. [69]	Thrissur	Microbial Inhibition Assay (*n* = 165)	8 (antibiotic residues)
		Charm Assay (*n* = 14)	21 (tetracycine)
		Charm Assay (*n* = 14)	28 (Beta lactam
		Charm ENRO (*n* = 14)	21 (Enrofloxacin)
Nirala et al. [70]	Bihar	HPLC (*n* = 250)	1.2 (Enrofloxacin)
Lunden [48]	Assam	Charm Rosa	88 (Neomycin, Streptocmycin)
			22.8 (Sulphonamide)
			2.9 (Beta lactams)
			2.3 (Chlorampenicol)
			2 (Macrolides / Gentamycin)
Lejaniya et al. [71]	Thrisssur	Antibiotic test kits (*n* = 50)	12 (Beta lactam)
			2 (Tetracyclines)
Gaurav et al. [72]	Punjab	Ridascreen competitive enzyme immunoassay (*n* = 133)	13.5 (Tetracycline)
Dinki and Balcha [67]	Guwahati city	*n* = 120	23.3 (type not specified)

In addition to the residue problem, several AMR resistant bacteria have been isolated in milk sampled within India. Kar et al. [74] reported ESBL producing *E. coli* in bovine milk from Odisha. Further, Koovapra et al. [75] reported ESBL producing *K. pneumonia* in bovine milk collected from three states of eastern and north-eastern India. More recently, ESBL producing *K. pneumonia* was isolated from buffalo milk and characterized [76]. All these studies pointed out that most of the ESBL producers from bovine milk also harboured AmpC type b-lactamase and plasmid mediated fluoroquinolone resistance gene(s). Sharma et al. [77] reported resistance to ampicillin, penicillin, nitrofurantoin (for *E. coli* isolates) and penicillin, cefotaxime, ampicillin, chloramphenicol, and tetracycline (for *S. aureus*). Oxytetracycline, streptomycin, ampicillin, and cloxacillin resistance was reported in the study by Verma et al. [39]. Methicillin-resistant *S. aureus*, methicillin-resistant *S. epididymis* and extended spectrum β- lactamase *E. coli* were reported from milk sampled from cows suffering from mastitis [78]. Further, vancomycin resistant *S. aureus* (VRSA) was reported for the first time in bovine and goat milk by Bhattacharyya et al. [79]; the use of vancomycin is important in human medicine.

### AMR interventions

The impact of AMR is known [80] and several mitigation approaches have been initiated or proposed, at various levels (Table 3). The Global Action Plan (GAP) on AMR, adopted by the World Health Assembly in 2015 [20] provides a broad framework of combating antimicrobial resistance. It has 5 strategic objectives which include improving awareness and understanding of AMR, strengthening knowledge through surveillance and research, reducing incidence of infectious diseases, optimizing the use of antimicrobial agents, and ensuring sustainable investments in countering AMR. GAP was supposed to guide member countries develop their own National Action plans (progress is assessed through annual monitoring of activities) [84]. The Global Antimicrobial Resistance Surveillance system (GLASS) was established in 2015 and the aim was to foster and strengthen national AMR surveillance, and to ensure production of reliable information [18]. The World Health Organization established a list of critically important drugs [85] whose use in animals should be restricted to help preserve their effectiveness. There has been a push for new drug development but emerging resistance hampers progress [80] and pharmaceuticals lack incentives to drive the process [86]. Although a challenge in developing countries, providing infrastructure to support diagnosis, establishing a continuous AMR surveillance system, and monitoring of interventions is essential [1, 80, 87]. Alternatives to antibiotic use, including the use of probiotics, bacteriophages (which cause bacterial lysis), and quorum-sensing inhibitors (which attenuates bacterial virulence) have been proposed [86, 88, 89].

**Table 3:** Suggested strategies to reducing antimicrobial usage in human and animal health, and conserving their effectiveness

Reference	Description of proposed AMR strategies
CDDEP [81]	Reducing the need for antibiotics use (improved water, sanitation, immunization); hospital infection control; change incentives that encourage antibiotic use to incentives that encourage antibiotic stewardship; reduce and eventually phase out antibiotic use in agriculture; educate and inform health professionals, policy makers, and the public on sustainable use of antibiotics. *https://www.cddep.org/wp-content/uploads/2017/06/swa_executive_summary_edits_2016.pdf*
CDDEP [4]	Tracking rates of veterinary antibiotic use, resistance and residues through a nationwide surveillance and monitoring system; changing incentives to discourage unnecessary antibiotic use in animals (without jeopardizing animal or human health); education of farmers, veterinarians, and consumers on the dangers of antibiotic resistance; and phasing out the sub-therapeutic use of antibiotics in animals *https://www.cddep.org/wp-content/uploads/2017/06/india_abx_report-2.pdf*
Garg and Mohanta [28]	Educating farmers and other stakeholders on appropriate use of antibiotics; reducing the need for antibiotics through good husbandry practices and use of alternative medicines (herbal, probiotics etc.); allowing for withdrawal period to pass before products are sold; and enacting of laws that ban or restrict the use of antibiotics in animals.
Parikh [63]	Education on rational use of drugs; regulate over-the-counter availability of drugs; develop guidelines at the local, national and regional levels; improved hygiene and infection control; regular surveillance of data and antibiograms to guide antibiotic selection; antibiotic stewardship; culture tests before antibiotics are administered; measuring outcomes to evaluate effectiveness of policies.
Ghafur et al. [8]	Ban on over- the-counter drug sales; expanding the network of accredited laboratories and developing low cost diagnostics; issuance of antibiograms at pre-defined intervals by microbiology laboratories; reduce erroneous reporting through use of standardized laboratories; establishment of national antibiotic resistance surveillance system; evaluate levels of use of antibiotics in animal health; observing drug withdrawal periods; and monitoring of AMR in food animals.
GARP [82]	Surveillance for both AMR and antibiotic use; increased use of diagnostic tools; strengthening of infection control committees; continuing education for pharmacists and health staff; checklists for surgical procedures; improving antibiotic supply chains and quality; regulate veterinary use of antibiotics (ban non-therapeutic use of antimicrobials and observance of drug withdrawal periods).
Lee et al. [83]	Initiation of internship programs for postgraduate students; education of healthcare professionals; reductions in the amount of antibiotics used in agriculture; and promotion of antimicrobial stewardship activities

Antimicrobial resistance is a priority issue in India and there has been several attempts to contain it, among them, the formulation of the national policy for AMR containment, the “Chennai Declaration”, the *“Jaipur declaration on Antimicrobial Resistance”*, National Action Plan on AMR of 2017, and the “Redline” campaign (Table 4). The National policy for containment of antimicrobial resistance [90] outlines a number of AMR-mitigation strategies, including monitoring the use and misuse of antibiotics, setting up of hospital-based surveillance systems to monitor antibiotic resistance, documenting prescription patterns and establishing antibiotic monitoring system, enforcement of regulatory provisions for human, veterinary and industrial uses, promoting rational antibiotic use, and strengthening of diagnostics. The “Chennai” declaration of 2012 was meant to formulate a road map to tackle AMR in India [8]. The *“Jaipur declaration on Antimicrobial Resistance”* was adopted in 2011, and among other things, health ministers of WHO Southeast Asia region agreed to institute a comprehensive and integrated national approach to combat AMR, formulate multi-sectoral national alliances against AMR, regulate the use of antimicrobial agents and increase capacity for efficient AMR surveillance [91]. The National programme on containment of AMR (2012–2017) was formed in 2011 to help establish an AMR surveillance system (with an initial target of 30 network laboratories), to strengthen infectious disease control guidelines, and promote rational use of antibiotics *(**https://ncdc.gov.in/**).* “Healthcare Associated Infections” (HAI) surveillance (HAI Surveillance) is a collaborative surveillance project of the “All India Institute of Medical Sciences” (AIIMS), New Delhi, the Centres for Disease Control and Prevention (CDC), and the Indian Council of Medical Research (ICMR) *(**https://www.haisindia.com/**).* “Antimicrobial Resistance Surveillance Research Network” (AMRSN) is a national network of antimicrobial resistance surveillance based on laboratory data from tertiary care academic centres [92]. It was launched in 2013 to, among other pathogens, explore resistance in *K. pneumonia*, *E. coli*, *S. enterica* and *S. aureus* [43]*.* The Indian Council of Agricultural Research (ICAR), with FAO’s support, has started a network programme on AMR surveillance in food animals and aquaculture, called INFAAR (Indian Network for Fishery and Animals Antimicrobial Resistance). The aim of the programme is to explore the resistance pattern of indicator and pathogenic bacteria isolated from food animals including fish. So far, 11 veterinary and animal science institutes and 8 fishery institutes have joined the network.

**Table 4:** Key steps in the regulation of AMR in India

Year when action was taken	Implementation details
2011	Adoption of the “Jaipur Declaration on Antimicrobial Resistance” by India’s health minister along with the health ministers of all member states of the WHO South-East Asia Region. They agreed to, among other things, institute measures to combat AMR, develop national antibiotic policy, regulate use of antimicrobial agents, promote behavioural change in prescribers and communities, build capacity for efficient surveillance of AMR, and strengthen diagnostic facilities.
2012	The “National Programme on Containment of Antimicrobial Resistance” was launched under the 12th five-year plan (2012–2017). AMR surveillance work started in 10 laboratories. A few guidelines were developed (national treatment guideline for antimicrobial use, guideline on infection control). A national Infection control policy is being finalized. An International Conference on AMR was organized in February 2016.
2016	A workshop “Combating Antimicrobial Resistance: A Public Health Challenge and Priority” was jointly organized by the Government of India and the WHO. The “Medicines with the Red Line” media campaign was launched.
2017	National network of veterinary laboratories for antimicrobial resistance (AMR) was established (the Indian Network for Fishery and Animals Antimicrobial Resistance (INFAAR)) *http://www.fao.org/india/news/detail-events/en/c/853974/*
2017	National action plan on AMR was adopted.
2017	Antibiotic Residue limits in meat was released by the Food Safety and Standards Authority of India (FSSAI)
2018	Kerala adopted the sub national State Action Plan *https://www.reactgroup.org/news-and-views/news-and-opinions/year-2018/kerala-india-launch-of-the-1st-sub-national-action-plan-on-amr/*
2019	Manufacture, sale and distribution of colistin and its formulations for food-producing animals, dairy, poultry, aqua farming and animal feed supplements prohibited *https://www.theweek.in/news/health/2019/07/22/Sale-of-antibiotic-Colistin-for-food-producing-animals-banned.html*

The National Health Policy of 2017 has a component on antimicrobial resistance which calls for; reduction in the over-the counter administration of drugs, restrictions on the use of growth promoters in animals, and pharmacovigilance [93]. India has a National Action Plan for AMR (NAP-AMR) (2017–2021) which has been developed in line with the Global Action Plan. Funding to undertake the proposed activities, and sustain them, mechanisms for efficient inter-sectoral coordination, effective implementation of regulations, and provision of technical stewardship across the country, have been identified as key challenges that could affect its implementation [68].

### Antimicrobial stewardship

Antimicrobial stewardship is less developed in Asia [94] and Singh [21] considers this as the main factor that is driving AMR in India (in addition to the blame on drug producers in the country). It refers to a set of actions that promote responsible use of antimicrobials [95]. The policy statement on antimicrobial stewardship by the Society for Healthcare Epidemiology of America (SHEA), the Infectious Disease Society of America (IDSA), and the Paediatric Infectious Disease Society (PIDC) defines antimicrobial stewardship as coordinated interventions designed to improve and measure the appropriate use of antimicrobial agents by promoting the selection of appropriate antimicrobial drug regimes including dosing, duration of therapy and route of administration [96]. Stewardship programs are executed by multidisciplinary teams who include experienced physicians, pharmacists, microbiologists, epidemiologists, and infectious disease specialists [83].

In a study in Spain, an antimicrobial consumption decrease (from 1150 defined daily doses (DDD) per 1000 occupied bed days to 852 doses) was reported in the study by Cisneros et al. [97], following an educational stewardship intervention. In another study, Timbrook and Hurst [98] reported a 16% reduction in DDD following a stewardship intervention (a 31% decrease in carbapenem use was also observed). In India, Singh et al. [99] reported a 14% monthly cost reduction and a compliance rate of 54% (*n* = 584). According to Walia et al. [100], many hospitals have infection control guidelines, however, antimicrobial agent prescription audit and feedback is only practiced by a small percentage of hospitals (30%). Although hospitals can report high adherence to hospital disease control guidelines, poor compliance can result from other aspects of antibiotic stewardship (e.g. in antibiotic prescription guidelines and usage surveillance) [92]. It is important for veterinarians to embrace the spirit of AMR stewardship [101] even though the initiative was first developed for health professionals. Parkunan et al. [59] interviewed a total of 106 veterinarians in India’s Haryana State and found 91% to be unaware of the term “antibiotic stewardship”. It is likely that strategies found to be successful in human health will also be successful in animal health [102]. The implementation of such should be preceded by awareness creation which, for instance, would require development of a national training plan that takes care of the various stakeholder needs [68].

### Theory of change (ToC) and its role in AMR mitigation

**Fig. 1:**
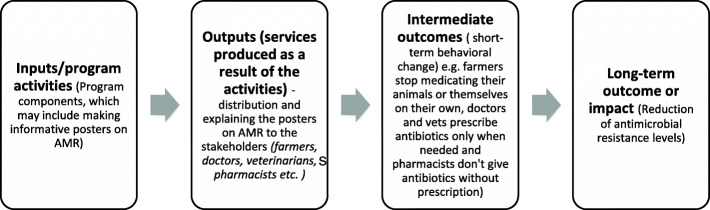
Sample theory of change for an AMR intervention

Antimicrobial resistance has gradually increased over the past two decades and is now widespread all over the world. It is also now clear that antibiotic over-use and misuse can contribute to emergence of antibiotic resistant bacteria. Several initiatives on AMR mitigation exist (or are proposed) hence the need to provide a clear demonstration of how each contributes to addressing the problem (which also allows for better use of resources). In a ToC, the long-term goal of a project is defined, and all the necessary pre-conditions are identified. Activities related to achieving each pre-condition (or early outcomes) are listed, and based on this, a program can decide on where to act given the available resources. A sample ToC map, for AMR mitigation is given in Fig. 1. Here, the connection between AMR interventions and the long-term outcome is shown. For each pre-condition, measurable indicators are defined and these aid in generating evidence in favour of an intervention (which is important in the realization of its long-term goal). Indicators can also be used in evaluation of AMR interventions. There are assumptions at each step of a theory of change (Table 5) from which appropriate research questions can be defined and tested.

**Table 5:** Sample assumptions in an AMR- intervention theory of change exercise

Input and project output	Outcome	Assumptions
Farmer receives training and written information on how to reduce antibiotic use and the importance of AMR	Farmers have increased knowledge on antibiotic use and AMR	• Farmers have enough background knowledge to understand the information • Farmers feel the relevance for them • Farmers are comfortable reading
Farmer receives messages, support and other communication that promote readiness to change	Farmers are motivated to change behaviour	• Farmers believe that change of behaviour will have benefits that exceed costs • Farmers believe that change of behaviour is feasible and socially desirable • Veterinarians and other actors stop promoting antibiotics
Farmers have access to options that can reduce antimicrobial use	Farmers change practice and reduce antibiotic use	• Farmers can afford inputs needed • Farmers can afford alternatives • Farmers see benefits from reducing antibiotics
	Reduction of antibiotics leads to reduced antimicrobial resistance in animals, animal products and animal environment	• There are no other sources of antibiotics for the animals that farmers cannot control • Reduced use per animal is not countered by increase in the number of animals
	Reduced AMR in humans	• AMR in animals is contributing significantly to human AMR

## Discussion

India is a leading producer and consumer of antibiotics [21, 103]. Food animals can contribute to AMR in several ways. Failure to use antimicrobials appropriately can cause bacteria to mutate and resist antibiotic treatment [104]. Other bacteria can acquire the resistance genes and also become resistant [105]. Antibiotics used in livestock are similar to those used by humans [8, 106], and AMR against drugs used in animal health has implications for public health. Mass medication of animals with critically important antimicrobials, either for therapy or prophylaxis, is a concern [19]. Once resistant, these bacteria can be passed to humans (through contaminated food, contact with the animals, or environment) [86]. The outcome is an infection which fails to respond to available drugs, necessitating the use of newer, perhaps more expensive antibiotics [105, 107]. Resistance to third generation cephalosporins could imply use of last resort carbapenems which may accelerate the problem of AMR [2, 4, 19].

Another important pathway for AMR emergence involves the release of drug residues in animal-source foods. Their presence, beyond the recommended levels, is an indication that antibiotics have been used and their withdrawal periods have not been adhered to [67]. Consumption of milk contaminated with the residues can result to allergic reactions [108] in addition to the public health effects. The residues, when present in milk, can also interfere with certain dairy processes (by inhibiting growth of lactic acid bacteria) [69, 109] which may also affect the quality of the product. Maximum Residue Limits (MRLs) for veterinary drugs in animal foods exist [15, 110]. For India, the CDDEP [4] report mentions a General Statutory Rule (GSR) 28 (E) which mandates a withdrawal period for antibiotics used in food animals. For some producers, discarding spoilt milk may not be practical given the economic loss incurred through the amounts rejected [23]; incentives that promote compliance while safeguarding farmer livelihoods need to be considered.

Antimicrobial resistance is complex and has many diverse causes [80]. In their paper on *“the pursuit of rational drug use: Understanding factors and interventions”*, Chauhan et al. [111] have outlined several factors that contribute to irrational drug use in human health, including aspects related to patients (demand for certain prescriptions, self-medication), drug suppliers (absence of regulations, drug promotion by pharmaceuticals), and prescribers (inadequate training, irrational prescriptions, faulty dispensing). In our review, we observed several practices that likely increase antibiotic usage in food animals, and consequently also add to the risk of AMR emergence and spread. Treatment of animals by farmers themselves and lack of proper recording makes it difficult to gather accurate data on antimicrobial use [1] which is an important aspect of AMR surveillance. A robust surveillance system is important in monitoring of AMR [43]. India has no nationwide database on AMU surveillance [1] and national burden of AMR is unknown [68]. Although quacks (i.e. informal animal health providers) seemingly help the community to access health care, especially in areas where professionals are not available, they are not trained, and their prescription practices could jeopardize on-going AMR-mitigation efforts. Inadequate professional personnel, which gives room for untrained individuals to offer animal health services, is not just a problem in India, as observed by Grace [112], animal treatments in sub-Saharan Africa are largely given by informal sector players who include the farmers themselves. Diagnostics including simple tests, although key in determining what antimicrobials to use, are often not performed, for reasons such as the lack of capacity [86] and their non-availability. The use of antibiotics in animals especially those that are important in human medicine should be restricted [28], in line with the recommendations of the World Health Organization [85]. Use of growth promoters in animal feeds is perceived to be common in India [4], and is worsened by the fact that farmers may not be aware that that purchased feeds have antibiotics in them [113]. Farmers are also often misled to buy antibiotics and add these to animal feeds and water [59]; out of 16 poultry farms studied by Brower et al. [114] in Punjab, 12 (67%) reported using antimicrobials for growth promotion. It is the public health risk of using antibiotics for growth promotion that led to the ban of growth promoters in the European Union in 2006 [115]. Colistin, a growth promoter initially meant for topical use in humans, is now being used in treatment of life threatening infections [19]. Development of resistance against this product would have serious implications in human health. In India, recommendations have been made to regulate the use of antimicrobials in animal feeds including strict monitoring of colistin use [4, 8, 116].

India has a few regulations on antibiotic use in food animals but implementation and compliance is an issue [4, 28, 94, 117]. Observing good husbandry practices can result to less clinical diseases and reduced need for antibiotic use in animals. Practices that increase animal production without reliance on non-therapeutic use of antibiotics need to be explored [118]. India has a rich ethno-veterinary medicine culture [119, 120], a tradition that is passed from one generation to the other [121]. Its use in mastitis control is being explored [36], which if successful and sustainable, can greatly reduce the on-farm use of antimicrobials. With the AMR problem, local herbs can be alternatives to antibiotic use [122], however, their seasonality and effectiveness concerns would need to be addressed.

Previous AMR-mitigation strategies focused on addressing gaps in health care systems (and not much on animal production and environment). It is important to recognize that AMR is a One Health issue and requires participation of all disciplines to achieve successful outcome. Existing materials (e.g. WHO guidelines on AMR) can be adapted for use in tackling AMR arising from animal treatment [123]. An intervention restricting the use of antibiotics in food animals yielded a 10–15% reduction in resistant bacteria [124]. Strategies to reduce over-the-counter sales should consider challenges of drug access to rural populations (who may not have any other access route) [9, 82]. Ghafur et al. [8] provided options for implementation of such bans, including a more liberal one where a few drugs are considered at the start of the intervention. The Indian NAP (for AMR) is a well-designed and comprehensive initiative, however, its implementation is slow mostly due to funding limitation; health is under state governments (which manage many other programs and may not afford to support newer initiatives) [125].

The ToC approach shows how program interventions link to specified long-term goals. AMR- reduction interventions vary-- can be educational, managerial, regulatory etc. [3, 111]. Awareness creation amongst all stakeholders has been listed as an objective in the Global Action plan intervention. Changing norms on antibiotic use would require a change in behaviour which may be influenced by a number of other factors [81]. The World Antibiotic Awareness Week aims to raise awareness about antibiotic resistance, and to encourage practices that would reduce emergence and spread of AMR*.* For many interventions, their impact on antimicrobial use [3] as well as on their ability to reduce AMR have not been evaluated. It is important to generate evidence to support the choice of interventions (which should also be designed in a manner that allows for their impact to be assessed) [3, 112].

## Conclusion

Antimicrobial use is a problem in India. Addressing the gaps identified in our review can contribute to AMR reduction, but how this happens (i.e. the change process) can be explored by engaging stakeholders in a theory of change exercise. Interventions that address AMR from the animal health perspective need to be encouraged and should be designed in a manner that allows for their monitoring and evaluation. Initiatives such as the National programme for containment of AMR, National Action Plan on AMR, and the National Health policy point to government’s commitment in addressing the problem of AMR in the country and should be supported. Measures that increase compliance by the various stakeholders need to be explored. In addition, incentives that encourage actors to change their practices and effect AMR interventions need to be explored.

## Data Availability

Not applicable.

## Abbreviations

AIIMS: All India Institute of Medical Sciences
AMR: Antimicrobial Resistance
AMRSN: Antimicrobial Resistance Surveillance Research Network
AMU: Antimicrobial Use
CDC: Centres for Disease Control and Prevention
CDDEP: Center for Disease Dynamics, Economics and Policy
DDD: Defined Daily Doses
ESBL: Extended Spectrum Beta-Lactamases
FAO: Food and Agricultural Organization of the United Nations
GAP: Global Action Plan
GARP: Global Antibiotic Resistance Partnership
GLASS: Global Antimicrobial Resistance Surveillance system
GSR: General Statutory Rule
HAI: Healthcare Associated Infections
ICAR: Indian Council of Agricultural Research
ICMR: Indian Council of Medical Research
IDSA: Infectious Disease Society of America
INFAAR: Indian Network for Fishery and Animals Antimicrobial Resistance
SHEA: Society for Healthcare Epidemiology of America
ToC: Theory of Change
PIDC: Paediatric Infectious Disease Society
WHO: World Health Organization
